# Characterization of hematopoietic stem cells from the canine yolk sac

**DOI:** 10.1590/1984-3143-AR2021-0012

**Published:** 2021-07-19

**Authors:** Bárbara Rossi de Sousa, Vanessa Cristina de Oliveira, Alessandra Oliveira Pinheiro, Carlos Eduardo Ambrósio

**Affiliations:** 1 Departamento de Medicina Veterinária, Faculdade de Zootecnia e Engenharia de Alimentos, Universidade de São Paulo, Pirassununga, SP, Brasil

**Keywords:** yolk sac, canine, hematopoietic, stem cell and embryo

## Abstract

The characterization of hematopoietic stem cells (HSC) from the canine yolk sac (cYS) can contribute to future gene therapies because it is possible to obtain information about the beginning of the development of the circulatory system through the characterization. The cYS is a likely source of HSC, which is a source of blood cell development in mammals. Studies in this field have been conducted for decades; however, interest in cellular therapy is currently at its peak with greater visibility, and these cells are a promising therapeutic tool for the treatment of diseases related to animals and humans. The aim of this study was to isolate and characterize HSC from the cYS embryos at 30 to 45 days of gestational age. Our results showed that the cYS was macroscopically located in the ventral region with a central portion and extremities. The cells in culture presented a circular morphology and cell clusters. The average cell viability was 22.55% dead cells out of 6.5 × 10^4^ total cells. The cells were also able to form colonies on methylcellulose. Flow cytometry analysis revealed the expression of CD34, CD117, and CD45. Our results suggest that the cYS can be used as a source of hematopoietic cells, and this study is very important to understand the mechanism and development of the hematopoietic system in dogs.

## Introduction

A yolk sac is present in all vertebrate embryos, and it corresponds to a structure that is connected to the ventral region of the embryo-shaped bag (Oliveira et al., 2017). In placental mammals, this is reduced when nutrition is provided via the placenta and produces the proteins required for development (Moore and Persaud, 2004; Santos and Azoubel, 1996).

Its permanence is distinct in different species. In swine, the yolk sac involution occurs around 20 days of gestation without forming the placenta; whereas, in ruminants, it is transiently present but degenerates shortly after deployment has started, such that it is visible within 25 to 50 days of gestation (Oliveira et al., 2017). In horses, the placenta forms from the yolk sac, which regresses around the 40th day of gestation (Hyttel et al., 2009). In primates, it degenerates, and it does not have this feature (Noden et al., 1990). In humans, the yolk sac is developed during the second week of gestation, connecting the ventral side of the embryo to the intestine and blood circulation (Jones and Jauniaux, 1995).

In canines, the yolk sac persists until birth, existing as an extra-embryonic membrane, which is wrinkled and highly vascularized (Lee et al., 1983; Bjorkman et al., 1989; Wenceslau et al., 2011), that can function as an important mediator of exchange between the embryo and the mother (Rüsse et al., 1992). Its function is complex, and it participates in hematopoiesis, which is the development of the first blood cells and a primitive part of the circulatory system, in addition to the transfer of maternal materials, such as amino acids, vitamins, and proteins (Riveros, 2009; Wolf et al., 2003; Palis and Yoder, 2001).

Thus, the yolk sac is likely to be a source of stem cells, which include the first blood cells during development in mammals, and erythrocytes, which express transcription factors that direct these cells to their hematopoietic destination (Shivdasani et al., 1995; Robb et al., 1995).

The formation of blood cells is related to the development of endothelial cells in the yolk sac, leading the hypothesis that both cell types originate from a common precursor: hemangioblast (Jaffredo et al., 2005). The origin of the hemangioblast was previously described in murine, at the time the first hematopoietic and vascular stem cells developed from the extra-embryonic mesoderm of the yolk sac at 7.5 days of gestation (Yokomizo et al., 2001). The first blood cells formed in the yolk sac are nucleated erythrocytes. When these erythrocytes mature they generate the enucleated erythrocytes that enter blood circulation. After that, the hematopoietic cells are found in the aorta-gonadal mesonephros region, yolk sac, liver and bone marrow (Mikkola and Orkin, 2006).

The stem cells of the fetal attachments have been previously characterized in humans and several animal species, and their uses have been demonstrated, due to their high plasticity and proliferation rate, differentiation capacity in cell groups, low immunogenicity and immunomodulation effects, and the possibility for the formation of storage banks. The uncovering of multiple populations of stem and stem-like cells, each with unique properties, enables their exploitability for current and future therapies (Ambrósio et al., 2011; Vidane et al., 2017; Cardoso et al., 2017; De Vita et al., 2013).

Many studies have been conducted that have examined stem cells, and apparently these studies are able to obtain plasticity. In canines, the presence of hematopoietic stem cells has been observed in the bone marrow and in the blood of the umbilical cord (Bruno et al., 1999; Bruno et al., 2001). Studies have shown that hematopoietic cells in canines have the reconstitution ability and plasticity of stem cells, which allows for the use of a canine model in various scientific and therapeutic proposals, because they can provide pre-clinical information (Nakage and Santana, 2006). Thus, knowledge of stem cells has developed, both in terms of the capacity expansion and differentiation (Herzog et al., 2003; Alves et al., 2007).

Hematopoietic cells from the yolk sac have previously been described in mice, sheep and cattle (Auerbach et al., 1996; Pessolato et al., 2012; Oliveira et al., 2017). All the studies showed that of the cells coming from the yolk sac is of fundamental importance to understand this primitive embryonic annex generator of an initial system, emphasizing the relation between functional of this organ with the embryo in formation. Therefore, the aim of this study was to characterize the hematopoietic stem cells from cYS collected at different gestational stages to elucidate their roles during primary hematopoiesis.

## Material and methods

### Laboratories, material collection, and procedures

The study protocol was approved by the research ethics committee (4598140116) of the Faculty of Animal Science and Food Engineering, University of São Paulo, Brazil. For the analysis of the hematopoietic stem cells from the yolk sac, canine embryos, around 30 to 45 days of gestational age, were used from the uteri of pregnant females without breed that originated from the castration program of the Veterinary Hospital at the University of Sao Paulo, Campus Pirassununga. The pregnant uteri were transported to the Laboratory for Cultivation and Cell Therapy of the Veterinary Medicine Department USP/FZEA, Pirassununga.

### Patterning of hematopoietic cells from a bovine yolk sac in culture

The fetal membranes (corion, allantodoid and amnion) were removed and the yolk sac was accessed. Then, the yolk sac (tissue) was placed on plates of 35 mm Petri dishes (Corning 430588, USA) and washed with phosphate-buffered saline-L (PBS-L) solution and then digested enzymatically with 0.5% collagenase IV (Sigma- C2674, St Louis, USA) for 1 h. After this process the cells (3 × 10^4^) were plated in 24-well plates and incubated at 37°C in 5% CO_2_ and a relative humidity close to 80%. After confluence around 80%, these cells were replicated in 12-well plates with culture medium.

### Cell viability

The cells cultured from the yolk sac culture, containing 8.5x10^4^ were centrifuged, and the cell pellet was resuspended in freezing medium composed of 70% Medium Stem Pro® 34-SFM supplemented with 20% SFB, 10% DMSO, and 1% streptomycin/penicillin. This was distributed in cryotubes (1 ml per cryotube); then, it was transferred to a Mister frozen device and kept in -80°C freezer overnight. After 24 h, the cryotubes were placed into liquid nitrogen, where they were stored at -196°C for later use. The cryotubes were carefully removed from the liquid nitrogen drum and defrosted in a water bath at 37°C. Then, the cells were transferred to Falcon tubes, and the culture medium, Stem 34 Pro® SFM (Serum Free Medium), was added. The samples were centrifuged at 6000× g for 5 min the supernatant was discarded, and the pellet was resuspended in 2 ml of culture medium and subsequently transferred with medium to cultivation plates that were placed at 37.0°C.

After 24 h, the cell viability was determined by counting the number of living cells compared to the number of dead cells in a Neubauer chamber with trypan blue (1:1) and were counted.

### Colony-forming assays

Colony-forming assays were performed with 1x10^5^cells plate in Stem Pro34 media. After 2 days *in vitro,* the cells were grown in semisolid Methocult H4434 Classic (Stem Cells Technology) human growth factor, and a matrix containing recombinant cytokines and other supplements (methylcellulose, fetal bovine serum (FBS), bovine serum albumin (BSA), 2-mercaptoethanol, stem cell factor, GM-CSF, IL-3, erythropoietin, supplements, and Iscove’s MDM). Colony formation in vitro was optimized over a period of 15 days, during which the colonies were stained with crystal violet and subsequently analyzed.

### Immunophenotypic characterization of yolk sac cells by flow cytometry

Approximately 10^5^ cells were distributed into tubes for flow cytometry analysis of each marker. The cells were washed with 1 ml of Dulbecco’s phosphate-buffered solution FACS buffer, containing 0.1% BSA, and centrifuged at 6000 ×g for 8 min. The cells were incubated with the primary antibody (1:100), for 30 min at room temperature to analyze the expression of each immune cell population. After incubation, the cells were washed once with 1 ml of FACS buffer to remove excess antibodies and were incubated with the secondary antibody (1:300) for 30 min. The cells were washed again with FACS buffer and fixed with a 4% buffered paraformaldehyde solution. Samples were analyzed using a flow cytometer (Attune, Applied Biosystems) equipped with two lasers (red and blue). The cell populations were estimated by evaluating the percentage of cells expressing each of the markers, relative to the total number of cells acquired. To accomplish this procedure, a panel of three antibodies and two secondary antibodies were used (Table 1).

**Table 1:** Primary/secondary antibodies used in the flow cytometry analysis of the yolk sac-derived hematopoietic cells from the canine embryos.

**Antibodies**	**Specificity**	**Isotype**	**Supplier**	**Catalog no.**
**Primary antibody**				
CC171- mouse	CD45	IgG2a	Santa Cruz Biot. (Dallas, TX, USA)	SC-101839
C-18- goat	CD34	IgG	Santa Cruz Biot. (Dallas, TX, USA)	SC-7045
YB5.B8- human	CD117	IgG1	Chemicon (Billerica, MA, USA)	MAB1162F
**Secondary antibody**				
FITC goat anti-mouse IgG (H&L)		IgG1	Dako	F0474
Alexa Fluor 488 Rabbit anti-goat IgG (H&L)		IgG	Molecular Probes (Eugene, OR, USA)	A11075

## Results

### Macroscopic and cell culture analyses

**Figure 1:**
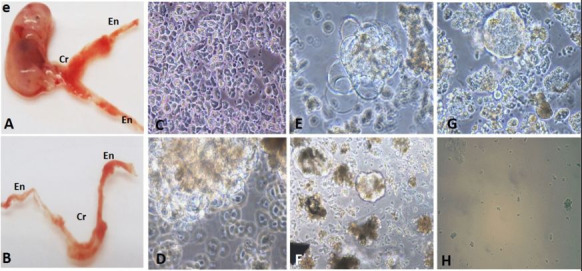
In (A) and (B) photography of cYSc; embryo (e), central region of yolk sac (Cr), ends of yolk sac (En). Photomicrograph of cells from cYS, in (C) and (D) cells with 20 days of cultivation; In (E) is observed a cell cluster formed. In (E), (F) and (G) note non-adherent cells with a circular shape; (H) note the change in cell morphology compared to the previous photomicrographs.

Macroscopically the cYS has a transparent membrane that was highly vascularized, the central portion which was localized in the ventral region of the embryo, and two extremities, and this was close to the umbilical cord (Figure 11B). The cells were non-adherent in culture and for the first 5 to 10 days a circular morphology was observed. During the period of 15 to 25 days, formation of grape-like cell clusters appeared. The cells remained in culture for 27 days, and after this period, the cells presented with reduced size and an irregular membrane presented a suggestive cell death (Figure 1CH).

### 3.2 Cell viability

Triplate cells samples for the cell viability test were defrosted after 1 week of cryopreservation, stained with Tripan Blue, and counted with the aid of a Neubauer chamber to obtain the values ​​shown in Table 2.

**Table 2:** List of cryopreserved cell values before and after defrosting.

**Dish**	**Number of cells frozen**	**% dead after desfrosting**	**% living after desfrosting**
**1**	8.5 × 10^4^	26.48%	73.52%
**2**	8.5 × 10^4^	23.53%	76.47%
**3**	8.5 × 10^4^	17.65%	82.35%
**Average**		22.56%	77.44%

**Figure 2:**
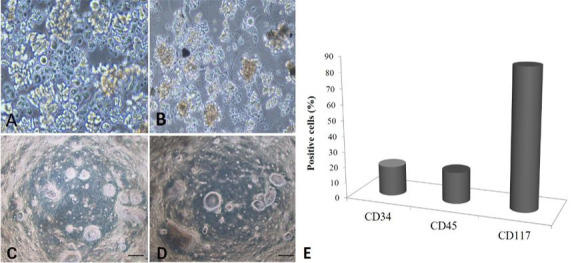
Cell viability, Colony-forming cells, and Immunophenotyping analyses. In (A) and (B) cell viability, A cell morphology before freezing; (B) after freezing; (C) and (D) colonies formed with a spherical shape; (E) note the positive expressions of CD34 (19%), CD45 (20%) and CD117 (88%).

After cell counting, the cells were plated and observed during successive passages to assess the potential for cell proliferation after the cryopreservation process. The cell viability analysis showed that HSC remained with the same characteristics after freezing (Figure 2A and B). In our experiments, cryopreservation of these cells was feasible, reaching a cell death rate of 22.56% after freezing.

### Colony assays (colony-forming cells; CFCs)

For the colony assays, the cells were plated on methylcellulose. Homogeneous and heterogeneous colonies were observed, considering cell diameter and individual morphology. The homogenous colonies were more centralized and showed a spherical aspect, with a high cell density (> 200 cells were observed in the colonies) (Figure 2C and D). Whereas, the heterogeneous colonies were dispersed at the peripheral portion of the plate, showed different morphologies, and had low cell densities

### Immunophenotypic characterization of the yolk sac cells by flow cytometry

The cells were cultured in Stem Pro-34 SFM and analyzed via flow cytometry, using a panel of three antibodies: CD34, CD45, and CD117 (c-kit).

In our experiments, we found expression of all of the markers, the cells were found to be immunopositive for CD34 (19%), CD45 (20%) and CD117 (88%) (Figure 2E).

## Discussion

The canine model represents a viable option that can substitute human models in certain circumstances because the physiology of dogs is more similar to humans when compared to other experimental models (Kirkness et al., 2003). Currently, there are no reports in the literature about the differentiation potential that these cells can present in dogs. Studies that use embryonic attachments are on the rise because of their potential as alternative sources of stem cells. Since these cells are relatively easy to obtain and isolate, *in vitro* studies are being carried out to increase basic knowledge regarding their origin and processing mechanisms, aiming to apply this knowledge in future clinical research and procedures.

The fetal membranes have been used in regenerative medicine and key to this is the uncovering of multiple populations of stem and stem-like cells, each with unique properties that can be exploited for future therapies. Also they are strong candidates for veterinary regenerative medicine because they exhibit high capacities for cellular differentiation (Lim, 2017; Ambrósio et al., 2011).

Due to the fact that the yolk sac is the primary site of formation of the blood and endothelial cells and because they have homogeneous, primitive morphology and because they do not express mature cell markers on their surface or even antigens encoded by the Major Histocompatibility Complex (MHC), cells from this tissue become an important source of stem cells for cell therapy (Medvinsky et al., 2008; Wilpshaar et al., 2002).

The YS is one of the extra-embryonic membranes that plays an important role in the initial survival of the embryo, as a source of nutrition during the period when the true placenta is not yet fully formed (Oliveira et al., 2017).

In the yolk sac, the development of the initial waves of hematopoiesis occurs, which provides the first functioning blood cells for the developing embryo. In mice, the yolk sac is essential for the development of the embryo, because it provides the initial feto-maternal transport system, before the placenta is formed, and is the source of the first blood cells (Ferkowicz and Yoder, 2005; Li et al., 2003).

Hematopoietic cells from the canine yolk sac were found to be similar to this from yolk sac bovine, sheep and mouse (Oliveira et al., 2017; Auerbach et al., 1996; Pessolato et al., 2012). Was possible observed in culture non-adherent cells with cluster formation.

In our study, the cryopreservation of cells was feasible, reaching a cell death rate of 22.55% after freezing, which is similar to the findings of hematopoietic cells from the yolk sac of sheep embryos (Pessolato et al., 2012).

Stem cells from human embryos and S17 murine bone marrow hematopoietic progenitor cells were subjected to a colony formation test, by culturing them in a semisolid media (typically methylcellulose or agar), supplemented with cytokines. In the methylcellulose assays, the cells produced, on average, CFCs after 14 days of co-culture, which was the maximal number of CFCs (Kaufman et al., 2001).

In our experiments, colonies with more 200 cells (15 days in culture) were observed, similar to the findings in human embryonic stem cells and S17 murine bone marrow hematopoietic cells (Kaufman et al., 2001). Our data were also similar to those for cells from the yolk sacs of bovine embryos, which exhibited CFCs in hematopoietic cells after 14 days of culture (Oliveira et al., 2017). Our results differed from those obtained with hematopoietic cells from the yolk sacs of sheep embryos, which demonstrated CFCs after 1 day of cultivation on methylcellulose (Pessolato et al., 2012).

Immunophenotypic characterization of the canine hematopoietic cells from the yolk sac was carried out by analyzing the expression of the markers CD34, CD45, and CD117.

CD34 (Cluster of Differentiation 34) is a glycosylated transmembrane protein (antigen) that is expressed in hematopoietic progenitor cells, in addition to endothelial, fibrotic, and nerve cells. It is located in the cell membrane, provides cellular adhesion, and has an important role as a marker for the identification of hematopoietic progenitor cells (Sutherland et al., 1993; Wang et al., 2016).

CD45 (protein tyrosine phosphatase) is a type C receptor that is expressed in all hematopoietic lines, except mature erythrocytes and platelets (Sasaki et al., 2001).

Recently Bian et al (2020) isolated cells from the yolk sac from human embryos. They noted that a yolk sac-derived parent population was among the first CD45 + hematopoietic cells to emerge.

CD117 (c-kit) is a receptor of the tyrosine kinase transmembrane factor of hematopoietic lineage that is encoded by the gene *kit1*, which is called the stem cell factor. It is a co-mitogen of hematopoietic stem cells and a differentiation factor for mast cells. Thus, it is a common marker for hematopoietic progenitors and is important in hematopoietic development (Miettinen and Lasota, 2005; Jindal et al., 2014).

CD117 was also expressed in mice; however, low expression of CD45 was observed, which is similar to our findings. The expression of these three markers demonstrates that the cells possess hematopoietic capacity (Li et al., 2003; Tárnok et al., 2010).

CD117 is expressed in vasculogenesis, and this expression suggests the presence of a common precursor for hematopoietic and endothelial cells, the hemangioblast (Palis and Yoder, 2001; Auerbach et al., 1996; Cumano et al., 2001).

The cells were found to be immunopositive for CD34 (19%), CD45 (20%), with a high expression of CD117 (88%), which was similar to the results found in bovine yolk sac at a different gestational age and number of days in culture (Li et al., 2003; Oliveira et al., 2017).

CD117 was also expressed in mice; however, low expression of CD45 was observed, which is similar to our findings. The expression of these three markers demonstrates that the cells possess hematopoietic capacity (Tárnok et al., 2010).

## Conclusion

We conclude that the cells from the cYS have hematopoietic characteristics because they formed cell clusters in culture and demonstrated expression of hematopoietic markers (CD34, CD45, and CD117) via flow cytometry analysis. Additionally, the cells were capable of forming colonies and could be cryopreserved without losing their viability. Further studies need to be carried out to validate their ability and later use in regenerative medicine. Our data is an initial study that suggests that the hematopoietic cells derived from the cYS provide an appropriate *in vitro* model for studying the biology of hematopoiesis.
